# On the Use of Acetone or Wood Naptha in Cholera

**Published:** 1849-08

**Authors:** Henry T. Child

**Affiliations:** Philadelphia


### *On the use of Acetone or Wood Naptha in Cholera.* By Henry T. Child, M. D., Philadelphia.

To the Editors of the Medical Examiner:

Gentlemen:—I beg leave to lay before you and the Profession, a few remarks in relation to the treatment of Cholera. During the last week I have had nine cases under care; and to illustrate the plan of treatment I will give an account of several cases.

The first was J. S., laboring man, aged about 40; strong constitution, and rather corpulent. He had had diarrhœa for five days, and on the 22d of last month, four days after the commencement of the diarrhœa, he walked about 20 miles. On the 24th I saw him, and prescribed a pill of camphor, opium and kino, which appeared to check the diarrhœa; but about 10 P. M., he was attacked with profuse watery diarrhœa and vomiting of "rice water," and when I saw him at one A. M. on the 25th inst. he had discharged between two and three gallons of serous fluid; the pulse was very small and corded; the hands and countenance shrivelled, eyes sunken. I gave him fifteen drops of acetone or wood naptha, and the vomiting, which had been incessant, ceased immediately, and did not return; he then had ten grains of calomel every hour for six hours. At this time the discharge from the bowels began to assume a greenish color and to diminish in quantity; the secretion of urine which had been arrested for twenty four hours returned, and he was convalescent; his recovery was rapid.

The second case was the Rev. H. C. Shelton of Ohio, aged 50, who was at a private boarding house in this city. He had diarrhœa for six hours before I was called to him. At this time it assumed the characteristic "rice water" appearance, and there was vomiting of a similar fluid. He took fifteen drops of the naptha and ten grains of calomel every hour—that is, one dose each half hour. The vomiting ceased immediately, and did not return; and in three hours he had bilious discharges, and the secretion of urine, which had been very much diminished, returned; in three days he was able to go out.

The third case was A. G., aged about 30—had had diarrhœa for two days; was under treatment of a physician. I was called in haste to see him. I found him in a profuse cold perspiration, no pulsation perceptible at the wrist. Countenance and limbs shrivelled, and already blue—in short, all the symptoms of fatal collapse. I gave him a *teaspoonful* of naptha, and in fifteen minutes gave fifteen grs. of calomel, with directions to repeat the dose every fifteen minutes, and was obliged to leave him. The next day I found him convalescent.

The fourth case, M. F., married, mother of four children, was delivered on the 27th inst. of a female child, and was very comfortable until the morning of the 30th, when she was suddenly attacked with watery diarrhœa and nausea. I gave her the naptha, in doses of ten drops, and calomel in five grain doses every hour. "Rice water" discharges continued for twenty-four hours, but the discharges assumed a green color, and she is now convalescent.

I am not prepared to explain the operation of the naptha, but as the result has been so satisfactory in all the cases that I have treated, I desire to call the attention of the profession to it. Of the calomel treatment little need be said, as the object is to restore the secretions as speedily as possible, and to equalize the circulation ; and it is probable we have nothing in the pharmacopœia that is so likely to produce these effects as calomel.

*Philadelphia, 7th mo. 2d,* 1849.

